# PYR/PYL/RCAR family members are major *in-vivo* ABI1 protein phosphatase 2C-interacting proteins in Arabidopsis

**DOI:** 10.1111/j.1365-313X.2009.04054.x

**Published:** 2009-11-09

**Authors:** Noriyuki Nishimura, Ali Sarkeshik, Kazumasa Nito, Sang-Youl Park, Angela Wang, Paulo C Carvalho, Stephen Lee, Daniel F Caddell, Sean R Cutler, Joanne Chory, John R Yates, Julian I Schroeder

**Affiliations:** 1 Division of Biological Sciences, Cell and Developmental Biology Section and Center for Molecular Genetics, University of CaliforniaSan Diego, 9500 Gilman Drive, La Jolla, CA 92093-0116, USA; 2 Department of Chemical Physiology, The Scripps Research Institute10550 North Torrey Pines Road, La Jolla, CA 92037, USA; 3 Howard Hughes Medical Institute and Plant Biology Laboratory, The Salk Institute for Biological Studies10010 N. Torrey Pines Road, La Jolla, CA 92037, USA; 4 Department of Botany and Plant Sciences, University of California–RiversideRiverside, CA 92521, USA

**Keywords:** abscisic acid, ABI1, ABI1 interacting proteins, proteome, PYR/PYL/RCAR

## Abstract

Abscisic acid (ABA) mediates resistance to abiotic stress and controls developmental processes in plants. The group-A PP2Cs, of which ABI1 is the prototypical member, are protein phosphatases that play critical roles as negative regulators very early in ABA signal transduction. Because redundancy is thought to limit the genetic dissection of early ABA signalling, to identify redundant and early ABA signalling proteins, we pursued a proteomics approach. We generated YFP-tagged ABI1 Arabidopsis expression lines and identified *in vivo* ABI1-interacting proteins by mass-spectrometric analyses of ABI1 complexes. Known ABA signalling components were isolated including SnRK2 protein kinases. We confirm previous studies in yeast and now show that ABI1 interacts with the ABA-signalling kinases OST1, SnRK2.2 and SnRK2.3 in plants. Interestingly, the most robust *in planta* ABI1-interacting proteins in all LC-MS/MS experiments were nine of the 14 PYR/PYL/RCAR proteins, which were recently reported as ABA-binding signal transduction proteins, providing evidence for *in vivo* PYR/PYL/RCAR interactions with ABI1 in Arabidopsis. ABI1–PYR1 interaction was stimulated within 5 min of ABA treatment in Arabidopsis. Interestingly, in contrast, PYR1 and SnRK2.3 co-immunoprecipitated equally well in the presence and absence of ABA. To investigate the biological relevance of the PYR/PYLs, we analysed *pyr1/pyl1/pyl2/pyl4* quadruple mutant plants and found strong insensitivities in ABA-induced stomatal closure and ABA-inhibition of stomatal opening. These findings demonstrate that ABI1 can interact with several PYR/PYL/RCAR family members in Arabidopsis, that PYR1–ABI1 interaction is rapidly stimulated by ABA in Arabidopsis and indicate new SnRK2 kinase-PYR/PYL/RCAR interactions in an emerging model for PYR/PYL/RCAR-mediated ABA signalling.

## Introduction

The plant hormone abscisic acid (ABA) controls important abiotic stress-induced and developmental responses, including maintenance of seed dormancy, seed development, growth regulation and stomatal closure. Forward and reverse genetic analyses have led to the identification of many loci encoding ABA signalling components that function in ABA metabolism and in ABA signalling (Finkelstein *et al.*, 2002; Nambara and Marion-Poll, 2005; Israelsson *et al.*, 2006; Yamaguchi-Shinozaki and Shinozaki, 2006). However, genetic redundancy has hampered the identification of many important ABA signalling components. Furthermore, proteins that mediate ABA reception are a subject of present debate and insufficient evidence exists on the interactions of some of the candidate proteins with known ABA signalling components (McCourt and Creelman, 2008).

Protein phosphorylation and dephosphorylation events are important mediators of ABA signal transduction. ABA-activated SNF1-related protein kinases 2 (SnRK2s) and Ca^2+^ dependent protein kinases (CDPKs) are positive transducers of ABA signalling (Li *et al.*, 2000; Mustilli *et al.*, 2002; Yoshida *et al.*, 2002; Mori *et al.*, 2006; Fujii *et al.*, 2007; Zhu *et al.*, 2007). Furthermore calcineurin B-like proteins (CBLs) and CBL-binding protein kinases (CIPKs), encode another class of SNF1-related protein kinases that negatively affect ABA responses (Hrabak *et al.*, 2003; Batistic and Kudla, 2009; Luan, 2009). The ABA-activated protein kinase OST1 (SnRK2.6/SnRK2E) functions as a positive regulator of ABA-induced stomatal closure (Mustilli *et al.*, 2002; Yoshida *et al.*, 2002). SnRK2.2 and SnRK2.3 are homologues of OST1 and are also activated by ABA and regulate ABA responses in seed germination, root growth and gene expression (Fujii *et al.*, 2007). Recently, *snrk2.2/2.3/2.6* triple mutants were shown to cause a strong ABA insensitive phenotype in seed germination, root growth and gene expression, suggesting that SnRK2.2, SnRK2.3 and OST1/SnRK2.6 have overlapping functions in ABA signalling (Fujii and Zhu, 2009; Nakashima *et al.*, 2009).

The dominant ABA insensitive mutants *abi1-1* and *abi2-1* exhibit ABA insensitivity in seed germination and root growth responses (Koornneef *et al.*, 1984). ABI1 and ABI2 encode type 2C protein phosphatases (PP2Cs) (Leung *et al.*, 1994, 1997; Meyer *et al.*, 1994; Rodriguez *et al.*, 1998). The Arabidopsis genome includes more than 76 *PP2C* genes (Schweighofer *et al.*, 2004; Xue *et al.*, 2008). Signal transduction analysis in guard cells showed that *abi1-1* impairs ABA signalling mechanisms including ABA activation of S-type anion channels (Pei *et al.*, 1997), ABA-induced [Ca^2+^] cyt elevations (Allen *et al.*, 1999), ABA-induced Ca^2+^ channel activation and ROS production (Murata *et al.*, 2001) and ABA activation of the OST1 protein kinase in Arabidopsis (Mustilli *et al.*, 2002). These findings led to the model that ABI1 PP2C functions very early in ABA signal transduction (Pei *et al.*, 1997; Allen *et al.*, 1999; Murata *et al.*, 2001; Mustilli *et al.*, 2002). These signal transduction studies prompted us to search for *in vivo* ABI1-interacting proteins via protein complex purifications in the present study to identify possible redundant early ABA signal transduction proteins.

Studies have shown that six of the nine Arabidopsis PP2Cs belonging to cluster A of the PP2Cs family (Schweighofer *et al.*, 2004), ABI1, ABI2, HAB1, HAB2, AHG1 and PP2CA/AHG3 function as negative regulators of ABA signalling and have independent and overlapping functions (Gosti *et al.*, 1999; Merlot *et al.*, 2001; Leonhardt *et al.*, 2004; Saez *et al.*, 2004, 2006; Kuhn *et al.*, 2006; Yoshida *et al.*, 2006b; Nishimura *et al.*, 2007; Rubio *et al.*, 2009). Yeast two hybrid screens have identified substrates of ABI1, including the homeodomain transcriptional factor ATHB6 (Himmelbach *et al.*, 2002), the protein kinases, CIPK15, CIPK20 and OST1 (Guo *et al.*, 2002; Ohta *et al.*, 2003; Yoshida *et al.*, 2006a), gluthathione peroxidase3 (Miao *et al.*, 2006) and a homologue of the yeast SWI/SNF chromatin-remodelling complex, SWI3B (Saez *et al.*, 2008).

Recently two independent groups have reported that two Bet VI family proteins, PYR1 and RCAR1, interact with ABI1, ABI2 and HAB1 (Ma *et al.*, 2009; Park *et al.*, 2009). These studies have provided evidence that the recombinant protein PYR1 and the RCAR1-ABI2 protein complex, may function as ABA receptors in ABA signalling (Ma *et al.*, 2009; Park *et al.*, 2009). Two dimensional NMR analyses of PYR1 showed that ABA causes shifts in the conformation of PYR1 (Park *et al.*, 2009). Furthermore, ABA stimulates the interaction of PYR1 with PP2Cs and this ABA-dependent interaction down regulates PP2C activity *in vitro* (Park *et al.*, 2009). RCAR1 also negatively regulates PP2C activity after ABA treatment *in vitro*, though RCAR1 constitutively interacts with PP2Cs. However, whether PYR/PYL/RCAR proteins interact with ABI1 in Arabidopsis remains unknown.

Here, to identify unknown redundant early ABA signalling proteins *in vivo*, we pursued YFP–ABI1 complex affinity column-based purifications from Arabidopsis plants. Interestingly, we identified nine of the 14 PYR/PYL/RCAR family members as the most robustly co-purified proteins with ABI1. These data show ABI1 interaction with multiple members of PYR/PYL/RCAR family in Arabidopsis.

Here we further address the following emerging questions: (i) Does the ABI1 protein phosphatase interact with the PYR1 protein *in vivo* (ii) Does ABA affect this interaction *in vivo* and within which time frame? (iii) Does ABI1 interact with SnRK2.2, SnRK2.3 and OST1/SnRK2.6 in plants? (iv) Does PYR form complexes with these ABA signalling SnRK2 kinases and does ABA affect this interaction? and (v) Do PYR/PYL/RCAR function in ABA-induced stomatal closure and ABA inhibition of stomatal opening?

## Results

### Isolation of YFP–ABI1 over-expression plants

**Figure 1:**
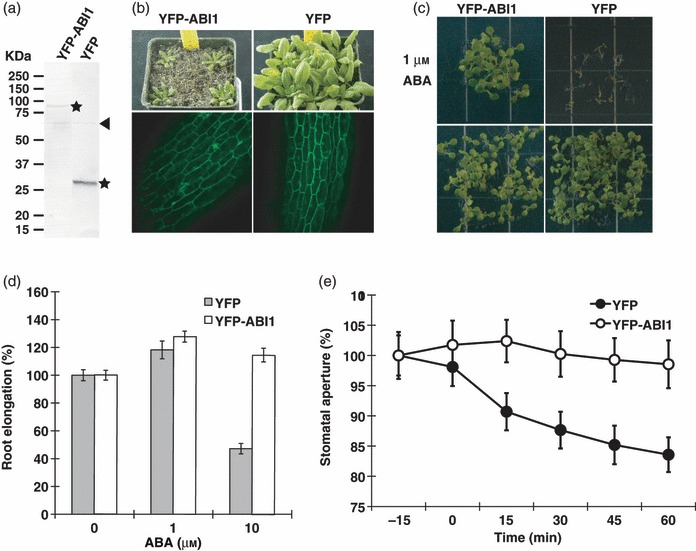
Constitutive expression of YFP–ABI1 causes ABA insensitivity. (a) Western blot analysis of YFP-tagged ABI1 (left) and YFP control (right) proteins in transgenic Arabidopsis. Stars indicate predicted YFP–ABI1 and YFP bands. Arrow head: non-specific band. (b) Morphology and subcellular localization of Arabidopsis plants constitutively expressing YFP–ABI1 (left) and YFP (right) at the rosette plant stage. Plants were grown for 5 weeks in soil. (c) ABA-insensitive phenotype of constitutively expressed YFP–ABI1 (left) and ABA response in control YFP-expressing seedlings (right). Top: in the presence of 1 μm ABA. Bottom: without added ABA. Transgenic plant seeds were sown on agar MS plates with or without 1 μm exogenous ABA. (d) ABA-dependent root growth responses of Arabidopsis seedlings constitutively expressing YFP and YFP–ABI1. Seedlings were germinated and grown on hormone-free MS plates for 5 days and then transferred to MS plates containing the indicated ABA concentrations. Root length was measured 4 days after transfer. Error bars show standard deviations. (e) Time course experiments of ABA-induced stomatal closing in YFP control and YFP–ABI1 expressing leaves (genotype blind experiments). Stomatal apertures were individually mapped and images captured (Siegel *et al.*, 2009) and measured before and after addition of 1 μm ABA. Average stomatal apertures at time −15 min: 3.47 ± 0.14 μm (YFP plants), 3.75 ± 0.18 μm (YFP–ABI1 plants). YFP plants: *n* = 23 stomata, YFP–ABI1 plants: *n* = 26 stomata. Error bars show SEM.

To assess further the ABA signalling cascade, we pursued experiments to identify ABI1-interacting proteins in Arabidopsis using affinity column-based protein complex purifications. We generated transgenic YFP–ABI1 and YFP Arabidopsis expression lines in an *abi1-3* knockout mutant background (Saez *et al.*, 2006). Five independent transgenic YFP–ABI1 expression lines were isolated. Western blot and fluorescence microscopy analyses showed that these Arabidopsis lines express the YFP–ABI1 and YFP proteins, respectively (Figure 1a,b). Interestingly, YFP–ABI1 expression plants showed growth and ABA insensitive phenotypes that were comparable to the dominant *abi1-1* mutant. 5-week-old YFP–ABI1 plants were significantly smaller in size than control YFP expression plants (Figure 1b). Previous research has reported that ABI1–GFP over-expressing lines do not show any ABA response phenotypes compared with vector control lines (Moes *et al.*, 2008). In our investigations, YFP–ABI1 fusion expression plants showed ABA insensitive phenotypes during seed germination, root growth and in stomatal responses compared with the control YFP expression plants confirming ABI1 function (Figure 1c–e).

### Identification of ABI1-interacting proteins

Using YFP–ABI1 and YFP expression in the *abi1-3* knock-out background, we purified ABI1-interacting proteins. A GFP affinity column was loaded with whole protein extracts from YFP–ABI1 and control YFP expression plants grown on MS plates for 21 days with or without ABA treatment. Affinity purified protein complexes were identified by mass spectrometric analyses. The specificity of the proteins purified by YFP affinity purification was analysed in parallel negative control experiments using YFP expression plants in the *abi1-3* mutant background (Tables S1 and S2). Upon silver staining, some visible bands overlapped with controls and specific bands associated that YFP–ABI1 samples were also consistently observed (Figure S1). Mass-spectrometrical analyses of five samples without ABA treatment (four independent samples and one duplicate) and five samples treated with ABA (three independent samples and two duplicates) allowed identification of proteins that associated with YFP–ABI1. Interestingly, the identified proteins included known ABA signalling components SnRK2.2, SnRK2.3, RPN10 and OST2/AHA1 (Tables 1, 2, S5 and S6) (Smalle *et al.*, 2003; Fujii *et al.*, 2007; Merlot *et al.*, 2007). SnRK2.2 and SnRK2.3 were not identified in any of the YFP control experiments (Table S2).

**Table 1:** Known ABA signalling proteins co-purified with ABI1 from Arabidopsis plants without addition of exogenous ABA

			Sequence coverage (%)					
			
Symbol	Locus	AGI	Ex1	Ex2	Ex3	Ex4	Ex5	Average
ABI1	AT4G26080.1	AT4G26080	90.6	87.1	83.2	87.1	87.6	87.1 ± 2.6
SnRK2.2	AT3G50500.1	AT3G50500	–	–	22.4	–	13.5	18.0 ± 6.3
SnRK2.3	AT5G66880.1	AT5G66880	–	–	8.9	12.5	16.6	12.7 ± 3.9
RPN10	AT4G38630.1	AT4G38630	10.6	–	14.0	7.3	11.7	10.9 ± 2.8
OST2	AT2G18960.1	AT2G18960	5.6	–	12.9	2.8	–	7.1 ± 5.2

**Table 2:** Known ABA signalling proteins co-purified with ABI1 from Arabidopsis plants exposed to exogenous ABA

			Sequence coverage (%)					
			
Symbol	Locus	AGI	Ex6	Ex7	Ex8	Ex9	Ex10	Average
ABI1	AT4G26080.1	AT4G26080	63.4	85.5	89.4	84.6	82.3	81.0 ± 10.2
SnRK2.2	AT3G50500.1	AT3G50500	13.5	16.6	26.0	–	–	18.7 ± 6.5
SnRK2.3	AT5G66880.1	AT5G66880	13.6	11.4	19.9	–	–	15.0 ± 4.4
RPN10	AT4G38630.1	AT4G38630	14.5	11.7	13.7	7.3	–	11.8 ± 3.2
OST2	AT2G18960.1	AT2G18960	–	3.9	10.3	–	–	7.1 ± 4.5

**Figure 2:**
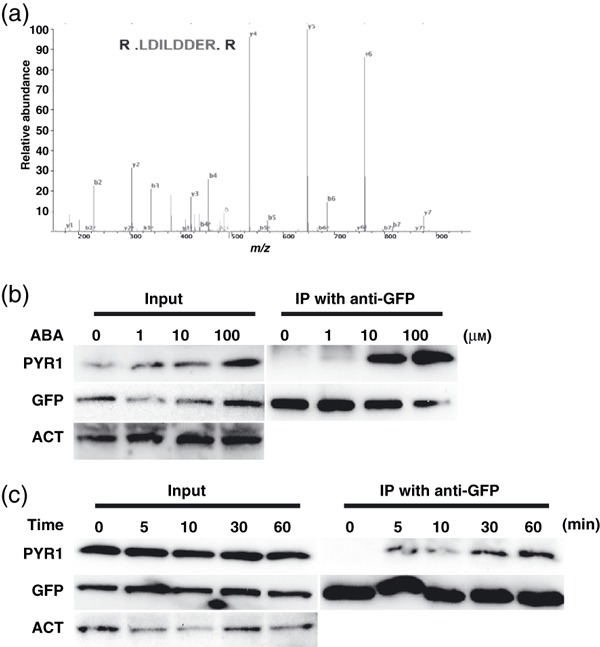
ABA causes ABI1–PYR1 interaction in Arabidopsis. (a) Example of raw tandem mass spectrometry data identifying PYR1 peptide in YFP–ABI affinity purified samples. The predicted PYR1 peptide sequence is shown in the top inset. (b) Increasing concentrations of exogenously applied ABA strongly enhanced co-immunoprecipitation of YFP–ABI1 with PYR1 in Arabidopsis. Transgenic Arabidopsis plants were exposed to ABA for 3 h. (c) ABA triggers interaction of YFP–ABI1 with PYR1 within 5 min of exposure of Arabidopsis plants to exogenous ABA. Left: Total protein extracts (left, Input) from YFP–ABI1 plants. Plants were grown on MS plates for 3 weeks and were treated with or without 100 μm exogenous ABA. After co-immunoprecipitation using anti-GFP beads, input and co-immunoprecipitated samples were detected with anti-PYR1 and anti-GFP antibodies.

Analyses of mass-spectra identified nine proteins in the absence of exogenously applied ABA that were identified in all YFP–ABI1 experiments, but not in YFP controls (Tables 3, S3, and S7). In the presence of exogenous ABA, eleven proteins were identified in all YFP–ABI1 samples (Tables 4, S4, and S8). Initially, we named these proteins candidate ABI1 interacting proteins (ABIP). The protein with the largest sequence coverage was ABI1 (Table 3). Six of the nine proteins in samples that were not treated with exogenous ABA encoded proteins from a, at the time uncharacterized, Bet VI subfamily of proteins (Tables 3 and S3). These Bet VI family proteins including PYR1 and other close homologues have recently been independently identified as candidate ABA-binding and ABA signalling proteins (Ma *et al.*, 2009; Park *et al.*, 2009). Interestingly the proteins co-purified with ABI1 with the highest total and unique sequence coverage and number of unique peptides and spectrum count were all members of the PYR/PYL/RCAR protein family (Figure 2a, Tables 3, S3 and S7). Two additional proteins from the 14 member PYR/PYL/RCAR protein family were identified in three or four of the five experiments (Table 3, PYL7 and PYL10). Moreover, in ABA-treated samples eight PYR/PYL/RCAR proteins had the largest total and unique sequence coverage and number of unique peptides and spectrum count of the isolated ABI1-interacting proteins and were identified in all five LC-MS/MS experiments (Tables 4, S4 and S8). As Bet VI proteins were the most significantly and consistently represented proteins from among all sequenced protein peptides in YFP–ABI1 samples, but not in YFP control samples (Table S2), these findings provide evidence that the ABI1 protein phosphatase can form complexes with several of the 14 PYR/PYL/RCAR Bet VI family members *in vivo* (Figure 2a, Tables 3, 4, S1, S3, S4, S7 and S8).

**Table 3:** Candidate ABI1-interacting proteins with the largest mass-spectrometrical sequence coverage from five LC-MS/MS experiments analyzing ABI1 complexes isolated from Arabidopsis in the absence of exogenous ABA

				Sequence coverage (%)					
				
	Locus	AGI	Symbol	Ex1	Ex2	Ex3	Ex4	Ex5	Average
ABI1	AT4G26080.1	AT4G26080	ABI1	90.6	87.1	83.2	87.1	87.6	87.1 ± 2.6
ABIP1	AT5G53160.2	AT5G53160	PYL8	78.2	63.3	79.8	61.2	64.9	69.5 ± 8.8
ABIP2	AT2G38310.1	AT2G38310	PYL4	87.9	72.5	55.1	40.6	62.8	63.8 ± 17.8
ABIP3	AT5G05440.1	AT5G05440	PYL5	74.4	63.5	71.9	35.5	57.1	60.5 ± 15.7
ABIP4	AT1G01360.1	AT1G01360	PYL9	67.9	50.3	44.9	62.6	59.9	57.1 ± 9.4
ABIP5	AT2G40330.1	AT2G40330	PYL6	57.7	22.3	48.4	28.4	34	38.2 ± 14.6
ABIP6	AT4G17870.1	AT4G17870	PYR1	39.8	19.4	36.1	28.8	48.2	34.5 ± 10.9
ABIP11	AT3G53430.1	AT3G53430	RPL12B	28.3	15.1	31.3	5.4	5.4	17.1 ± 12.3
ABIP12	AT5G20280.1	AT5G20280	ATSPS1F	9.2	4.9	9.4	5	4	6.5 ± 2.6
ABIP7^a^	AT4G01026.1	AT4G01026	PYL7	46.9	–	37	25.1	26.1	33.8 ± 10.3
ABIP8^a^	AT4G27920.1	AT4G27920	PYL10	–	–	15.3	7.7	7.7	10.2 ± 4.4
ABIP9^a^	AT5G46790.1	AT5G46790	PYL1	22.6	–	–	–	–	22.6 ± 0.0

a Three additional PYR/PYL/RCAR family members identified in 4, 3 and 1 of the experiments, respectively.

**Table 4:** Candidate ABI1-interacting proteins with the largest mass-spectrometrical sequence coverage from five LC-MS/MS experiments analyzing Arabidopsis ABI1 complexes exposed to exogenous ABA

				Sequence coverage (%)					
				
	Locus	AGI	Symbol	Ex6	Ex7	Ex8	Ex9	Ex10	Average
ABI1	AT4G26080.1	AT4G26080	ABI1	63.4	85.5	89.4	84.6	82.3	81.0 ± 10.2
ABIP2	AT2G38310.1	AT2G38310	PYL4	50.7	67.6	83.1	83.1	61.8	69.3 ± 14.0
ABIP6	AT4G17870.1	AT4G17870	PYR1	71.2	73.3	70.7	62.8	42.9	61.2 ± 12.5
ABIP1	AT5G53160.2	AT5G53160	PYL8	62.2	71.8	69.7	50	42	59.1 ± 12.8
ABIP9	AT5G46790.1	AT5G46790	PYL1	55.2	70.6	73.3	41.2	18.1	51.7 ± 22.8
ABIP4	AT1G01360.1	AT1G01360	PYL9	27.3	77	61.5	45.5	32.6	48.8 ± 20.6
ABIP3	AT5G05440.1	AT5G05440	PYL5	30	43.3	83.3	42.4	34.5	46.7 ± 21.2
ABIP7	AT4G01026.1	AT4G01026	PYL7	12.3	64.9	69.7	49.3	17.5	42.7 ± 26.6
ABIP5	AT2G40330.1	AT2G40330	PYL6	32.1	42.3	52.1	30.7	23.3	36.1 ± 11.2
ABIP13	AT3G19390.1	AT3G19390	MLD14.3	13.7	21.9	7.3	13.3	13.3	13.9 ± 5.2
ABIP14	AT2G20580.1	AT2G20580	AtRPN1a	2.8	10.7	10.1	5.6	5.6	7.0 ± 3.3
ABIP8^a^	AT4G27920.1	AT4G27920	PYL10	–	7.7	–	–	–	7.7 ± 0.0

a PYL10 was identified in one experiment as shown.

### ABI1 interacts with PYR1 in Arabidopsis

It was recently shown that ABA enhances the interaction of YFP–ABI1 with HA–PYR1 transiently expressed in *Nicotiana benthamiana* leaves (Park *et al.*, 2009). To assess whether these interactions occur *in vivo* in Arabidopsis, we performed co-immunoprecipitation experiments in stably transformed YFP–ABI1 and YFP control expression lines using a PYR1 antibody. YFP–ABI1 interacted with PYR1 in Arabidopsis with a strong enhancement upon application of 100 μm exogenous ABA for 48 h (Figure S2). Subsequently, co-immunoprecipitation experiments were performed three h after exogenous application of 0, 1, 10 and 100 μm ABA. ABA caused a clear enhancement in ABI1–PYR1 interaction *in vivo* (Figure 2b). To determine the timing of this ABA response in Arabidopsis, co-immunoprecipitation experiments were performed 0, 5, 10, 30 or 60 min after application of exogenous ABA. These experiments showed a clear ABA induction of a YFP–ABI1 and PYR1 interaction within 5 min of ABA treatment *in vivo* (Figure 2c).

### PP2Cs, PYR1 and SnRK2s interact with each other in ABA signalling

**Figure 3:**
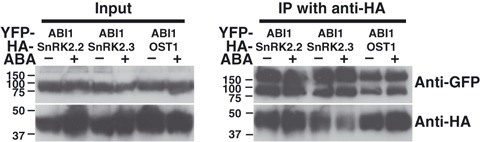
ABI1 PP2C co-immunoprecipitates with all three ABA signalling SnRK2 kinases in plants. YFP–ABI1 co-immunoprecipitates with HA–SnRK2.2, HA–SnRK2.3 and HA– OST1/SnRK2.6 both in the presence and absence of exogenously applied ABA. Total protein extracts (left, Input) from transformed *Nicotiana benthamiana* leaves were harvested 3 days after inoculation and were treated with or without 100 μm ABA for 24 h before harvesting. After co-immunoprecipitation using an anti-HA matrix, input and immunoprecipitated samples were detected with anti-GFP and anti-HA antibodies.

ABI1 has been reported to interact with OST1/SnRK2E/SnRK2.6 in yeast (Yoshida *et al.*, 2006a). However it remains unknown whether this interaction occurs in plant cells and whether ABI1 interacts with other SnRK2s. It also remains unknown whether any ABI1–SnRK2 interactions are regulated by ABA similar to the ABI1–PYR1 interaction. As SnRK2.2 and SnRK2.3 were co-purified with ABI1 in our affinity column purification experiments (Tables 1 and 2), we co-expressed YFP–ABI1 with either HA–SnRK2.2, HA–SnRK2.3 or HA–OST1/SnRK2.6 in *N. benthamiana* and performed co-immunoprecipitation experiments. YFP–ABI1 constitutively interacted with HA–SnRK2.2, HA–SnRK2.3 and HA–OST1/SnRK2.6 both in the absence and presence of exogenously applied ABA (Figure 3).

**Figure 4:**
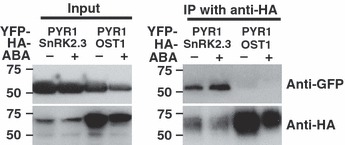
Interaction of PYR1 with SnRK2.3 and OST1. YFP–PYR1 co-immunoprecipitates with HA–SnRK2.3. In contrast HA–OST1/SnRK2.6 did not co-immunoprecipitate with YFP–PYR1. Total protein extracts (left, Input) from transformed *Nicotiana benthamiana* leaves were harvested 3 days after inoculation and were treated with or without 100 μm ABA for 24 h before harvesting. After co-immunoprecipitation using an anti-HA matrix, input and immunoprecipitated samples were detected with anti-GFP and anti-HA antibodies.

We analysed whether PYR1 might co-purify with SnRK2s. We co-expressed YFP–PYR1 with either HA–SnRK2.3 or HA–OST1/SnRK2.6 in *N. benthamiana* and performed co-immunoprecipitation experiments. Interestingly, YFP–PYR1 co-immunoprecipitated with HA–SnRK2.3 both in the presence and absence of ABA, but not detectably or very weakly with HA–OST1/SnRK2.6 (Figure 4).

### *pyr1/pyl1/pyl2/pyl4* quadruple mutant plants exhibit ABA insensitive phenotype in guard cells

**Figure 5:**
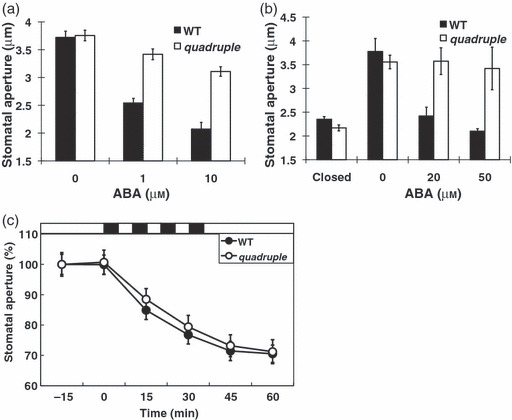
ABA-induced stomatal closure and ABA-inhibition of stomatal opening but not Ca^2+^ -induced stomatal closure are impaired in *pyr1pyl1pyl2pyl4* quadruple mutant plants. (a) ABA-induced stomatal closure in *pyr1pyl1pyl2pyl4* quadruple mutant and wild type rosette leaves treated with the indicated ABA concentrations for 1 h (*n* = 4 experiments, 30 stomata per experiment and condition; genotype and [ABA] blind experiments). (b) ABA inhibition of stomatal opening in *pyr1pyl1pyl2pyl4* quadruple mutant and wild type abaxial leaf epidermes treated with the indicated ABA concentrations for 1 h (*n* = 3 experiments, 30 stomata per experiment and condition; genotype and [ABA] blind experiments). Closed means incubation in the dark for 3 h (before light and ABA treatment). (c) Stomatal closure in response to four repetitive extracellular Ca^2+^ pulses (5 min each) is not impaired in *pyr1pyl1pyl2pyl4* quadruple mutant suggesting that PYR/PYL/RCARs function upstream of Ca^2+^. Four 5-min extracellular applications of 1 mm CaCl_2_ and 1 mm KCl were sequentially applied to abaxial leaf epidermes (black bars at top) followed by 50 mm KCl and 0 added CaCl_2_ exposure (White bars at top) (*n* = 31 individually mapped stomata for both *wild type* and *pyr1pyl1pyl2pyl4* quadruple mutant) Average stomatal apertures at time = −15 min: 5.24 ± 0.19 μm (wild type), 5.52 ± 0.14 μm (*pyr1pyl1pyl2pyl4* quadruple mutant). Error bars show SEM.

The *abi1-1* protein impairs ABA signalling upstream of known early ABA responses in Arabidopsis guard cells (Pei *et al.*, 1997; Allen *et al.*, 1999; Murata *et al.*, 2001; Mustilli *et al.*, 2002). A *pyr1/pyl1/pyl2/pyl4* quadruple mutant was recently shown to cause strong ABA insensitivities in seed germination, root growth and ABA-induced gene expression (Park *et al.*, 2009). Whether *PYR/PYL/RCAR* genes mediate ABA response in guard cells remains unknown. Public microarray data show that the *PYR1*, *PYL1*, *PYL2*, *PYL4*, *PYL5*, *PYL7* and *PYL8* mRNAs are highly expressed in guard cells and seedlings (Leonhardt *et al.*, 2004; Goda *et al.*, 2008; Yang *et al.*, 2008) (Figure S4). Stomatal response analyses were performed with *pyr1/pyl1/pyl2/pyl4* quadruple mutant plants. *pyr1/pyl1/pyl2/pyl4* quadruple mutants showed a strong ABA insensitive phenotype in double-blinded ABA-induced stomatal closing and ABA inhibition of stomatal opening analyses (Figures 5a,b and S5). Studies have provided evidence that Ca^2+^ signalling functions downstream of the *abi1-1* and *abi2-1* PP2Cs in guard cells (Allen *et al.*, 1999; Murata *et al.*, 2001). Experiments were pursued to determine whether *pyr1/pyl1/pyl2/pyl4* quadruple mutant plants impair Ca^2+^-induced stomatal closing. Repetitive addition and removal of Ca^2+^ to the extracellular solution bathing leaf epidermes while shifting the K^+^ equilibrium potential in guard cells, results in Ca^2+^-induced stomatal closure (Allen *et al.*, 2001). Such imposed extracellular Ca^2+^ pulses caused similar Ca^2+^-reactive and Ca^2+^-programmed stomatal closure responses in both *pyr1/pyl1/pyl2/pyl4* quadruple mutant and wild type epidermes (Figure 5c). These results suggest that these PYR/PYL/RCAR proteins are critical for ABA signalling in guard cells upstream of Ca^2+^ signalling.

## Discussion

Studies have suggested that ABI1 functions upstream of all analysed events in early ABA signal transduction in guard cells (Pei *et al.*, 1997; Allen *et al.*, 1999; Murata *et al.*, 2001; Mustilli *et al.*, 2002). We hypothesized that redundant proteins may act upstream of ABI1, because forward genetic screens had not identified other upstream loci. Our early attempts to co-purify ABI1-complexed proteins from Arabidopsis, showed that additional purification steps led to loss of many ABI1-associated proteins (I. Mori & J. I. Schroeder unpublished data). A rapid purification method using GFP affinity column-affinity purification, as reported here, was more fruitful for isolating redundant ABA signalling proteins *in vivo* (Tables 1–4, S1–S4, S7, S8). Here we identify nine of the 14 PYR/PYL/RCAR gene family proteins as *in vivo* ABI1-co-purified proteins with the largest sequence coverage and number of unique peptides and spectrum count of all identified proteins (Tables 3, 4, S3, S4, S7 and S8; Figures 2 and 3). After identifying these Bet VI family proteins as the main co-purified proteins in ABI1 complexes from Arabidopsis, we learned that PYR1 had been independently isolated in a chemical genetics screen as a candidate ABA signalling protein (Park *et al.*, 2009). In the absence of ABA, PYR1 and PYL4 proteins were co-purified as ABI1-interacting proteins (Tables 3, S3 and S7), although ABA enhanced the interaction of ABI1–PYR1 (Figure 2) and HAB1–PYL4 (Park *et al.*, 2009). Co-purification results in the absence of exogenously applied ABA may be due to the known endogenous ABA levels in Arabidopsis plants (Nambara and Marion-Poll, 2005). On the other hand, ABI1 seems to interact constitutively with PYL5 to PYL9 in Arabidopsis, in the absence of ABA, based on yeast two hybrid data (Ma *et al.*, 2009; Park *et al.*, 2009). Over-expression of several other PP2Cs causes ABA insensitive phenotypes in seed germination stages (Saez *et al.*, 2004; Kuhn *et al.*, 2006; Yoshida *et al.*, 2006b; Nishimura *et al.*, 2007). YFP–ABI1 expression plants showed ABA insensitive phenotypes during seed germination, root growth and in stomatal responses compared with the control YFP expression plants (Figure 1c–e).

In addition to the identification of PYR/PYL/RCAR proteins as the major proteins co-purified with ABI1 from Arabidopsis plants, the present study reports several further relevant new findings: (i) A new PYR1 antibody was used and showed that the ABI1 protein phosphatase interacts with the PYR1 protein *in vivo* (ii) ABA stimulates this interaction in Arabidopsis within 5 min of ABA exposure. (iii) ABI1 co-immunoprecipitates with the SnRK2.2, SnRK2.3 and OST1/SnRK2.6 protein kinases in plants, extending previous yeast two hybrid findings (Yoshida *et al.*, 2006a). (iv) PYR1 forms complexes with these ABA signalling SnRK2 kinases in plants both in the presence and absence of ABA. (v) *PYR/PYL/RCAR* genes are critical for ABA-induced stomatal closure and ABA inhibition of stomatal opening.

### Protein phosphorylation and dephosphorylation events in ABA signalling

The protein kinase OST1/SnRK2.6 is phosphorylated within 5 min of ABA application (Mustilli *et al.*, 2002; Yoshida *et al.*, 2006a). ABA binds to PYR1 and RCAR1–ABI2 thus down-regulating PP2C activities (Ma *et al.*, 2009; Park *et al.*, 2009). In addition, *PYR/PYL/RCAR* genes are required for ABA activation of the SnRK2 kinases (Park *et al.*, 2009). Here ABA is shown to cause PYR1–ABI1 interaction in Arabidopsis, whereas PYR1 forms a constitutive complex with the SnRK2.3 kinase both in the absence and presence of ABA (Figure 4). Whether PYR1 directly binds to SnRK2s remains to be determined. Furthermore, ABI1 constitutively interacts with the SnRK2.2, SnRK2.3 and OST1/SnRK2.6 kinases *in planta* (Figure 3). These co-immunoprecipitation results support the hypothesis that these major ABA signalling components, PYR/PYL/RCARs, PP2Cs and SnRK2s, may form an ABA signalosome complex, rather than forming a simple sequential PYR1→ABI1→SnRK2 signalling pathway. An initial sequential model (Park *et al.*, 2009) would predict that SnRK2 kinases may only enter into a complex with PYR1 after ABA exposure.

One of the substrates of SnRK2s is a b-ZIP type transcription factor ABRE/ABF (Furihata *et al.*, 2006; Fujii *et al.*, 2007). Arabidopsis *ost2* and *slac1* mutants show strong ABA insensitive phenotypes in guard cells and act downstream of the OST1 kinase (Li *et al.*, 2000; Merlot *et al.*, 2007; Negi *et al.*, 2008; Vahisalu *et al.*, 2008). We isolated OST2/AHA1 as a candidate ABI1-interacting protein (Tables 1 and 2), which would be consistent with the model that the ABA signalling PP2Cs regulate multiple downstream signalling proteins (Pei *et al.*, 1997; Allen *et al.*, 1999; Murata *et al.*, 2001; Furihata *et al.*, 2006; Kuhn *et al.*, 2006; Nishimura *et al.*, 2007; Saez *et al.*, 2008;).

### PYR/PYL/RCAR family members regulate ABA signalling in guard cells

Several ABA receptors have been reported to impair ABA-induced stomatal closure; CHLH was reported as an ABA receptor but only one stable and available genetic allele cch (Mochizuki *et al.*, 2001) showed a strong ABA insensitive phenotype (Shen *et al.*, 2006). Recently, double mutants in the candidate ABA receptors, GTG1 and GTG2, were shown to cause ABA insensitivity in seed germination, root growth, stomatal movements and ABA-induced gene expression (Pandey *et al.*, 2009). For ABA-induced stomatal closure, *gtg1gtg2* double mutants exhibited a limited insensitivity to ABA compared with WT, suggesting that additional ABA receptors are needed to perceive ABA and transduce the ABA signal independently. More recently, the PYR/PYL/RCAR family proteins were identified as new ABA binding and signalling proteins (Ma *et al.*, 2009; Park *et al.*, 2009). Here we show that *pyr1/pyl1/pyl2/pyl4* quadruple mutant plants exhibit a strong ABA insensitive phenotype in ABA-induced stomatal closing and ABA inhibition of stomatal opening but not in Ca^2+^-induced stomatal closing (Figure 5), suggesting that PYR1/PYL1/PYL2/PYL4 function upstream of Ca^2+^ in ABA signalling and likely also upstream of Ca^2+^-independent signalling (Allan *et al.*, 1994; Siegel *et al.*, 2009). Furthermore PYR1/PYL1/PYL2/PYL4 mediate ABA signalling not only in seed germination and seedling stages (Park *et al.*, 2009) but also in guard cells (Figure 5).

In conclusion, *in vivo* affinity column purifications of ABI1 complex proteins provide evidence that the PYR/PYL/RCAR family of proteins are the most abundant and robust *in vivo* ABI1-complexed interactors in Arabidopsis. ABA rapidly stimulates PYR1–ABI1 interaction in Arabidopsis and additional findings indicate the possible assembly of an early ABA signalosome.

## Experimental procedures

### Plant materials and growth conditions

The *Arabidopsis thaliana* (L.) Heynh. ecotype Columbia (Col) was used in this study. Plant growth conditions, seed germination and root elongation assays were performed as described previously (Nishimura *et al.*, 2004; Mori *et al.*, 2006). ABA-induced and repetitive Ca^2+^ pulse-stomatal movement responses and apertures were analysed as described previously (Vahisalu *et al.*, 2008). For ABA inhibition of stomatal opening, to pre-close stomata, intact leaf epidermes attached to a cover slip were pre-incubated in buffer (10 mm KCl, 7.5 mm iminodiacetic acid and 10 mm MES/Tris, pH 6.2) for 3 h in the dark and exposed to the indicated ABA concentrations in the light (100 μmol m^−2^ s^−1^).

### Cloning and construction of transgenic YFP–ABI1 Arabidopsis lines

The full length ABI1 cDNA was cloned into pDONR vector (Invitrogen, http://www.invitrogen.com) and sequenced and then transferred to the destination vectors pH35YG and pEarleyGate 201 by Gateway LR recombination reaction (Invitrogen). *Agrobacterium tumefaciens* strain GV3101 was used to transform the *abi1-3* T-DNA insertion disruption line and wild-type Col-0 with plants by the floral dip method (Clough and Bent, 1998). Transgenic plant lines were screened for hygromycin resistance and homozygous T3 lines were isolated.

### Affinity column purification of YFP–ABI1 complexed proteins

Identification of proteins by mass spectrometry is described in the Experimental procedures S1.

### Co-immunoprecipitation in Arabidopsis and *Nicotiana benthamiana*

For protein extractions, 3-week-old seedlings (1 g) grown on horizontal MS plates were incubated for 2 h in water before ABA treatment. Co-immunoprecipitations were performed using Dynabeads Protein G (Invitrogen) captured with anti-GFP antibody (Abcam, http://www.abcam.com) according to the manufacture’s specifications. Co-immunoprecipitations in *N. benthamiana* were performed as described previously without modifications (Park *et al.*, 2009).

## SUPPORTING INFORMATION

**Figure S1.** Identification of ABI1-interacting proteins in Arabidopsis.

**Figure S2.** ABA-induced interaction between ABI1 and PYR1 in Arabidopsis.

**Figure S3.** Tandem mass spectrum of peptides from proteins ABIP8 and ABIP11 listed in Tables S3 and S4 that showed one unique sequence to the corresponding proteins.

**Figure S4.** Transcription profiles of ABI1, PYR1/PYLs and SnRK2s in seedlings, guard cells and mesophyll cells.

**Figure S5.** ABA-induced stomatal closure isimpaired in *pyr1pyl1-pyl2pyl4* quadruple mutant plants.

**Table S1.** List of candidate ABI1-interacting proteins in YFP–ABI1 plants derived from LC-MS/MS data.

**Table S2.** List of candidate YFP-interacting proteins in control YFP plants derived from LC-MS/MS data.

**Table S3.** The number of unique and total peptides and spectrum count of candidate ABI1-interacting proteins co-purified with ABI1 from Arabidopsis plants in the absence of exogenous ABA.

**Table S4.** The number of unique and total peptides and spectrum count of candidate ABI1-interacting proteins co-purified with ABI1 from plants exposed to exogenous ABA.

**Table S5.** The unique sequence coverage of known ABA signalling proteins co-purified with ABI1 from Arabidopsis plants without addition of exogenous ABA.

**Table S6.** The unique sequence coverage of known ABA signalling proteins co-purified with ABI1 from Arabidopsis plants exposed to exogenous ABA.

**Table S7.** The unique sequence coverage of candidate ABI1-interacting proteins co-purified with ABI1 from Arabidopsis plants in the absence of exogenous ABA.

**Table S8.** The unique sequence coverage of candidate ABI1-interacting proteins co-purified with ABI1 from plants exposed to exogenous ABA.

**Experimental procedures S1.**

Purification of YFP–ABI1 interacting proteins by affinity column purification methods.

Multidimensional protein identification technology (MudPIT).

Analysis of tandem mass spectra.

PYR1 expression and antiserum preparation.

Construction of the erecta+ *pyr1/pyl1/pyl2/pyl4* quadruple mutant.
