# Supplementary material for: PYR/PYL/RCAR family members are major in-vivo ABI1 protein phosphatase 2C-interacting proteins in Arabidopsis
Source: Plant J. 2009 Nov 9;61(2):290–9. doi: 10.1111/j.1365-313X.2009.04054.x (PMC2807913; doi:10.1111/j.1365-313X.2009.04054.x)
Supplement: Supplementary file 1 [file tpj0061-0290-SD1.doc]

**Supporting Information Methods**

**Nishimura et al.**

**Purification of YFP-ABI1 interacting proteins by affinity column purification methods.**

For protein extraction, 150 to 700 3-week-old seedlings (10 to 70 gFW) grown on MS medium-containing agar plates were incubated for 2 hours in water (-ABA samples). After pre-incubation for 2 hours in water the seedlings were treated with 100 μM ABA treatment for 48 hours (+ABA samples). Samples were ground to a powder in liquid nitrogen and resuspended into 2 X times extraction buffer (50 mM Na-phosphate (pH 7.4), 150 mM NaCl, 0.1 % NP-40, 1 mM DTT and 1 x protease inhibitor cocktail (P9599; Sigma); 20 to 140 mL). Crude extracts were then centrifuged at 20,000 g for 30 min at 4 °C. The complete supernatant was passed through Miracloth (Calbiochem) and Sterile Miller Filter Unit (0.45 μm; Millipore) and loaded onto an anti-GFP conjugated 1 mL HiTrap NHS-activated HP column (GE Healthcare). Anti-GFP affinity columns were generated according to the manufacture’s instructions (GE Healthcare). After a 50 to 100 mL wash with wash buffer (50 mM Na-phosphate (pH 7.4), 150 mM NaCl, 0.1 % NP-40), YFP control and complexed ABI1 interacting proteins were eluted into 10 mL elution buffer (0.3 M Glycine-HCl pH 3.0, 1 mM DTT and 1 x protease inhibitor cocktail (Sigma)), and were fractionated each (0.5 mL per fraction) and were TCA precipitated. Subsequently, each fraction was analyzed by silver staining and fractions for mass spectrometry analyses were selected. A 60 µL buffer solution (8 M Urea, 100 mM Tris, pH 8.5) was added to a TCA precipitated protein pellet to solubilize proteins. To reduce the mixture, 0.3 µL of 1 M TCEP was added (for a final concentration of 5 mM TCEP) and incubated at room temperature for 20 minutes. Iodoacetamide (1.2 µL) was added to alkylate (10 mM final concentration) samples. The samples were subsequently incubated at room temperature in the dark for 15 min. Endoproteinase Lys-C (0.1 µg/µL) was then added in the amount of 1.0 µL and shaken for 4 hours while incubated in the dark at 37 ºC. The Urea was diluted to 2M by adding 180 µL of 100 mM Tris pH 8.5. To obtain a final concentration of 1 mM CaCl_2_, calcium chloride (100 mM) was then added (2.4 µL). The addition of trypsin (0.5 µg/µL) in the amount of 4.0 µL followed. The resulting mixture was then shaken for 12 hours and incubated in the dark at 37ºC. Formic acid (90 %) was added (15.0 µL) (final concentration of the mixture to 5% formic acid). Samples were then centrifuged for 30 min at 2ºC on a table top centrifuge (Cociorva *et al.* 2006).

**Multidimensional Protein Identification Technology (MudPIT):**

Following digestion, proteins were pressure-loaded onto a fused silica capillary desalting column containing 3 cm of 5-µm strong cation exchange (SCX) followed by 3 cm of 5-µm C18 (reverse phase or RP material) pressure packed into a non-deactivated 250-µm inner diameter (i.d.) capillary. To complete sample assembly a 100-µm i.d capillary consisting of a 10-µm laser pulled tip packed with 10 cm 3-µm Aqua C18 material (Phenomenex, Ventura, CA) was attached to the filter union (desalting column–filter union–analytical column). The resulting split-columns were placed in-line with a ThermoFinnigan Surveyor MS Pump (Version 2.3; Palo Alto, CA) and analyzed using a customized 4-step separation method (90, 120, 120, and 150 min respectively) (Diop *et al.* 2008).

Step 1 utilized only buffer A (95 % water, 5 % acetonitrile, and 0.1 % formic acid) and buffer B (80 % acetonitrile, 20 % water, and 0.1 % formic acid). It began with 5 min of 100 % Buffer A, followed by the following buffer B gradients: 5 min of 0-10 %, 40 min of 10-45 %, and 10 min of 45-100 %. Twenty minutes of 100 % buffer B treatment followed and the gradient program ended with 10 min of 100 % buffer A. Steps 2-4 utilized Buffers A, B, and C (500 mM ammonium acetate, 5 % acetonitrile, and 0.1 % formic acid). Steps 2 and 3 each began with: 3 min of 100 % buffer A, a 1 min gradient of 0 – X % buffer C, 7 min of X % buffer C, a 1 min gradient of 0 – 100 % A, a 3.2 min gradient from 0 – 10 % buffer B, a 74.8 min gradient from 10 - 45% buffer B, and then a 5 min 45 - 100 % buffer B gradient. Ten minutes of 100% buffer B and a 5 min 0 - 100 % gradient of buffer A followed. The sequence ended with 10 min of 100 % buffer A. Buffer C portions consisted of 20 % for step 2 and 50 % for step 3.

Step 4 began in a similar fashion: 3 min of 100 % buffer A, a 1 min gradient of 0 - 100 % buffer C, 7 min of 100 % buffer C, a 1 min gradient of 0 - 100 % buffer A and a 8 min gradient from 0 – 10 % buffer B. Its 10 - 45 % buffer B gradient lasted for 85 min and the 45 - 100 % buffer B gradient was for 10 min. Ten minutes of 100 % buffer B and then a 15 min gradient of 0 – 100 % buffer A ensued with the run ending with 10 min of 100 % buffer A.

As a result of increasing the salt concentration (buffer C), the peptides will subsequently dissociate from the SCX and then with a gradient of increasing hydrophobicity (buffer B) elute from the RP into the ion source. A distal 2.5 kV spray voltage was applied to elute the peptides from the micro capillary column. This applied voltage caused the peptides to directly electro-spray into an LTQ 2-dimensional ion trap mass spectrometer (ThermoFinnigan, Palo Alto, CA). For each step of the multidimensional cycle one full-scan mass spectrum (400 - 2000 m/z) was acquired followed by 5 data-dependent MS/MS spectra at a 35 % normalized collision energy. The above-described HPLC solvent gradients and MS functions were all controlled by the Xcalibur data system Version 1.4 (Diop*, et al.* 2008).

**Analysis of Tandem Mass Spectra:**

As each step was executed, mass spectra were recorded to a RAW file. These data were then converted into .ms2 format through the use of RawXtract (Version 1.9.3). The remaining MS/MS spectra were searched with the SEQUEST™ algorithm (Eng *et al.* 1994) against the TAIR Arabidopsis thaliana protein database. Protein sequences from the database were reversed (decoy database) to assess the false positive rate (Peng *et al.* 2003). A computer cluster consisting of 100 1.2 GHz Athlon CPUs was used to perform the search (Sadygov *et al.* 2002)***.*** Enzyme specifity was not employed during the SEQUEST search and the final data set was filtered using the DTASelect™ (version 2.0.31h) program (Tabb *et al.* 2002; Cociorva *et al.* 2006). The digestion method analyzed was specified (--trypstat for tryptic digests) to specifically filter for peptides with trypsin specificity. A user-specified false positive rate was used to dynamically set XCorr and DeltaCN thresholds through quadratic discriminant analysis (MacCoss *et al.* 2002). This dataset was then further filtered to remove contaminants (i.e. keratin) through the use of the -e contaminant (excludes protein names matching contaminant) and -l keratin (excludes protein descriptions matching keratin) commands. The minimum filter criteria (2 peptides and half tryptic status) were utilized for the row datasets coupled in Tables S1 (YFP-ABI1) and S2 (YFP control).

**PYR1 expression and antiserum preparation**

Recombinant 6X-HIS- PYR1 protein was prepared as previously described (Park *et al.* 2009). Production of antiserum against 6X-HIS-PYR1 was contracted to R.B. Sargeant (Ramona, CA). 100 μg 6X-HIS-PYR1 was injected in complete Freund’s adjuvant into two rabbits, and boosted twice with 100 μg at 3-week intervals. The quality of antiserum after 3rd boost was tested by immunoblot and the final bleed was prepared. Crude antiserum was used in immunoblots and detected a single band corresponding to the expected size of PYR1 in imbibed seed protein samples.

**Construction of the *erecta*^+^ *pyr1/pyl1/pyl2/pyl4* quadruple mutant.**

The previously described quadruple mutant was constructed from publicly available mutants and a *pyr1-1* allele. This quadruple mutant contains *pyl4*, *erecta***^-^** in *pyl2* linked in cis, because the *pyl2-1* allele was in the Ler background. To isolate a recombinant lacking the *erecta* mutation, the homozygous quadruple strain (*pyr1-1*; *pyl1-1*; *pyl2-1*; *er*^-^; *pyl4-1)* was crossed to the homozygous *erecta*^+^ triple mutant (*pyr1-1*; *pyl1-1*; *er*^+^*; pyl4-1)*. ~1200 seeds of the self-progeny of this F1 were screened for strong ABA insensitivity using germination assays on plates containing 0.7 μM (+)-ABA. ~200 of the strongest ABA insensitive lines were transferred to soil and scored as adults for the *erecta* phenotype. 51 *erecta*^+^ plants were then genotyped gene specific primers shown below and *pyr1-1* was validate by sequencing PCR product:

LBb1 (5’-GCGTGGACCGCTTGCTGCAACT-3’) for *Pyl1,*

DS3-2 (5’-CGATTACCGTATTTATCCCGTTC-3’) for *Pyl2,*

LB2 (5’-GCTTCCTATTATATCTTCCCAAATTACCAATACA-3’) for *Pyl4*

and gene specific primers as below.

Pyr1 (+); 5’-ATCATATGC CTTCGGAGTT AACACCAGA-3’

Pyr1 (-); 5’-ATGAATTCACGTCACCTG AGAACCACTTC-3’

Pyl1 (+); 5’-AAAAAAGCCCGGGCAATGGCGAATTCAGAGTCCT-3’

Pyl1 (-); 5’-AAAAAAGTCGACTTACCTAACCTGAGAAGAGTT-3’

Pyl2 (+); 5’-AAAAAAGAATTCATGAGCTCATCCCCGGCCGTGA-3’

Pyl2 (-); 5’-AAAAAAGTCGACTTATTCATCATCATGCATAGGT-3’

Pyl4 (+); 5’-AAAAAAGAATTCATGCTTGCCGTTCACCGTCCT-3’

Pyl4 (-); 5’-AAAAAAGTCGACTCACAGAGACATCTTCTTCTT-3’

**References**

**Cociorva, D., Tabb, D.L. and Yates, J.R., 3rd** (2006) Validation of Tandem Mass Spectrometry Database each Results using DTASelect. *Current Protocols in Bioinformatics*, 13.14.11-13.14.14, supplement 16, John Wiley and Sons.

**Diop, S.B., Bertaux, K., Vasanthi, D., Sarkeshik, A., Goirand, B., Aragnol, D., Tolwinski, N.S., Cole, M.D., Pradel, J., Yates, J.R., 3rd, Mishra, R.K., Graba, Y. and Saurin, A.J.** (2008) Reptin and Pontin function antagonistically with PcG and TrxG complexes to mediate Hox gene control. *EMBO Rep*, **9**, 260-266.

**Eng, J., MacCormack, A. and Yates, J.R., 3rd** (1994) An Approach to Correlate Tandem Mass Spectral Data of Peptides with Amino Acid Sequences in a Protein Database. *J Am Soc Mass Spectrom*, **5**, 976-989.

**MacCoss, M.J., Wu, C.C. and Yates, J.R., 3rd** (2002) Probability-based validation of protein identifications using a modified SEQUEST algorithm. *Anal Chem*, **74**, 5593-5599.

**Park, S.Y., Fung, P., Nishimura, N., Jensen, D.R., Fujii, H., Zhao, Y., Lumba, S., Santiago, J., Rodrigues, A., Chow, T.F., Alfred, S.E., Bonetta, D., Finkelstein, R., Provart, N.J., Desveaux, D., Rodriguez, P.L., McCourt, P., Zhu, J.K., Schroeder, J.I., Volkman, B.F. and Cutler, S.R.** (2009) Abscisic acid inhibits type 2C protein phosphatases via the PYR/PYL family of START proteins. *Science*, **324**, 1068-1071.

**Peng, J., Elias, J.E., Thoreen, C.C., Licklider, L.J. and Gygi, S.P.** (2003) Evaluation of multidimensional chromatography coupled with tandem mass spectrometry (LC/LC-MS/MS) for large-scale protein analysis: the yeast proteome. *J Proteome Res*, **2**, 43-50.

**Sadygov, R.G., Eng, J., Durr, E., Saraf, A., McDonald, H., MacCoss, M.J. and Yates, J.R., 3rd** (2002) Code developments to improve the efficiency of automated MS/MS spectra interpretation. *J Proteome Res*, **1**, 211-215.

**Tabb, D.L., McDonald, W.H. and Yates, J.R., 3rd** (2002) DTASelect and Contrast: tools for assembling and comparing protein identifications from shotgun proteomics. *J Proteome Res*, **1**, 21-26.

**References**

**Goda, H., Sasaki, E., Akiyama, K., Maruyama-Nakashita, A., Nakabayashi, K., Li, W., Ogawa, M., Yamauchi, Y., Preston, J., Aoki, K., Kiba, T., Takatsuto, S., Fujioka, S., Asami, T., Nakano, T., Kato, H., Mizuno, T., Sakakibara, H., Yamaguchi, S., Nambara, E., Kamiya, Y., Takahashi, H., Hirai, M.Y., Sakurai, T., Shinozaki, K., Saito, K., Yoshida, S. and Shimada, Y.** (2008) The AtGenExpress hormone and chemical treatment data set: experimental design, data evaluation, model data analysis and data access. *Plant J*, **55**, 526-542.

**Leonhardt, N., Kwak, J.M., Robert, N., Waner, D., Leonhardt, G. and Schroeder, J.I.** (2004) Microarray expression analyses of Arabidopsis guard cells and isolation of a recessive abscisic acid hypersensitive protein phosphatase 2C mutant. *Plant Cell*, **16**, 596-615.

**Yang, Y., Costa, A., Leonhardt, N., Siegel, R.S. and Schroeder, J.I.** (2008) Isolation of a strong Arabidopsis guard cell promoter and its potential as a research tool. *Plant Methods*, **4**, 1-15.
