# Supplementary material for: PYR/PYL/RCAR family members are major in-vivo ABI1 protein phosphatase 2C-interacting proteins in Arabidopsis
Source: Plant J. 2009 Nov 9;61(2):290–9. doi: 10.1111/j.1365-313X.2009.04054.x (PMC2807913; doi:10.1111/j.1365-313X.2009.04054.x)
Supplement: Supplementary file 2 [file tpj0061-0290-SD2.pdf]

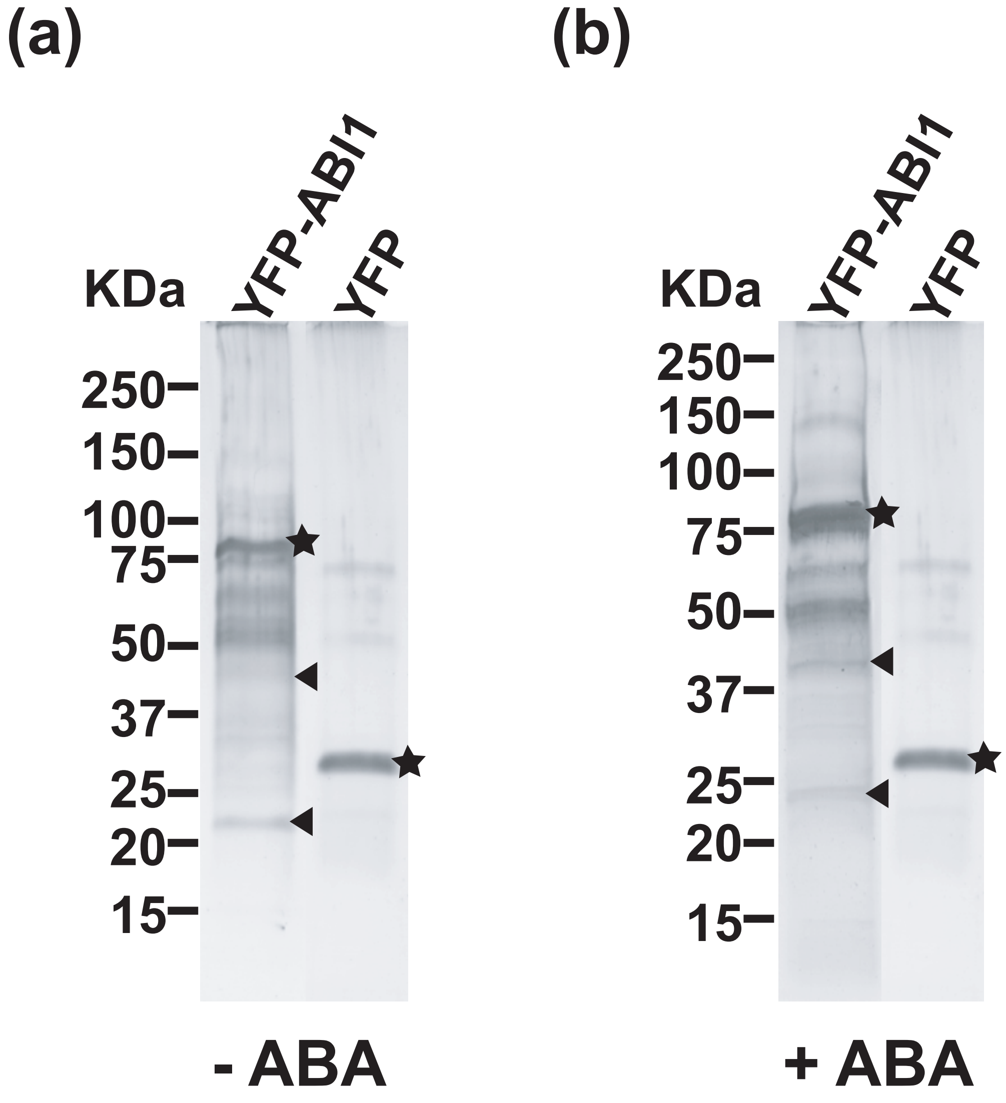

Figure. S1 Nishimura et al.

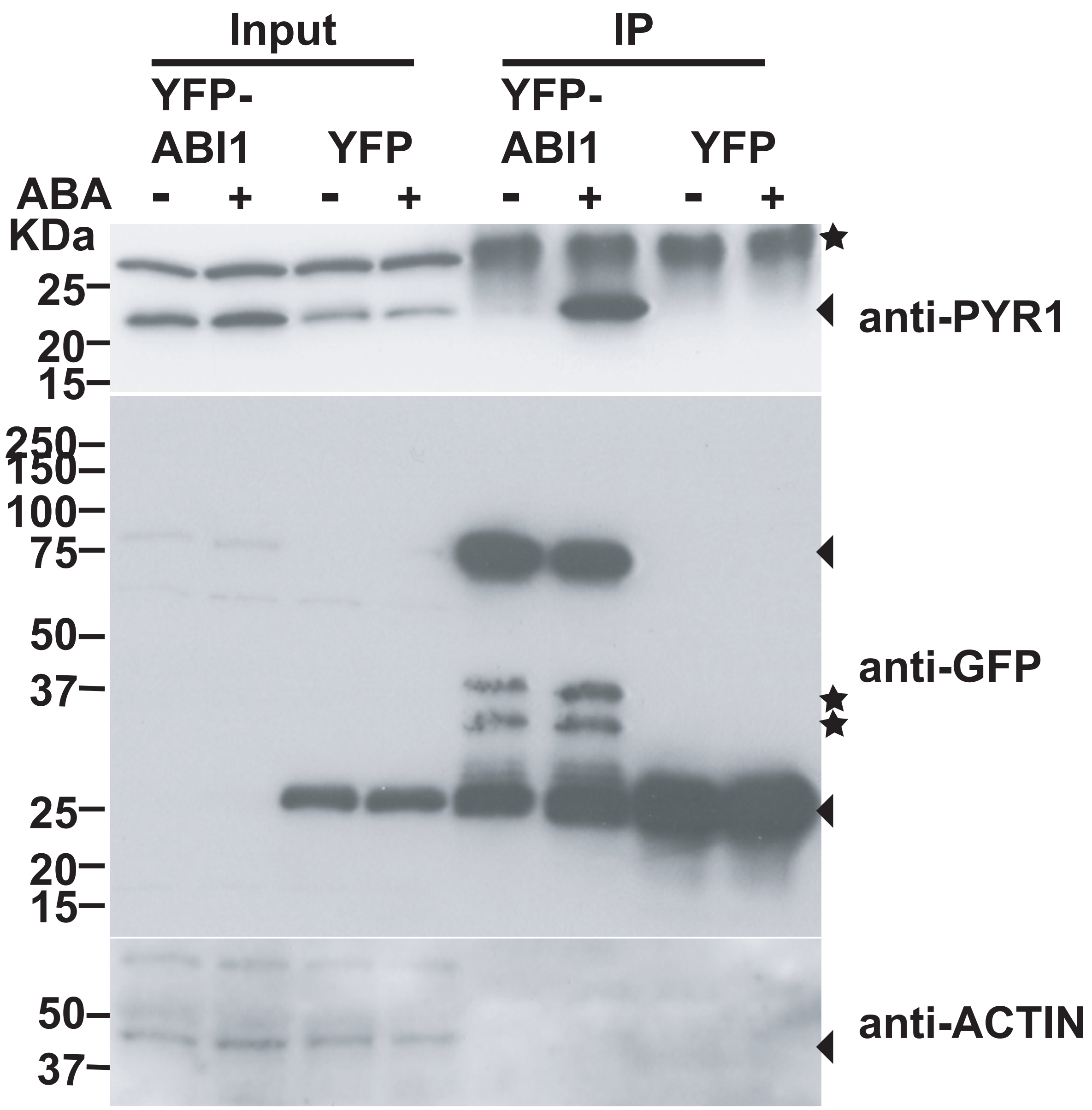

Figure. S2 Nishimura et al.

(a)

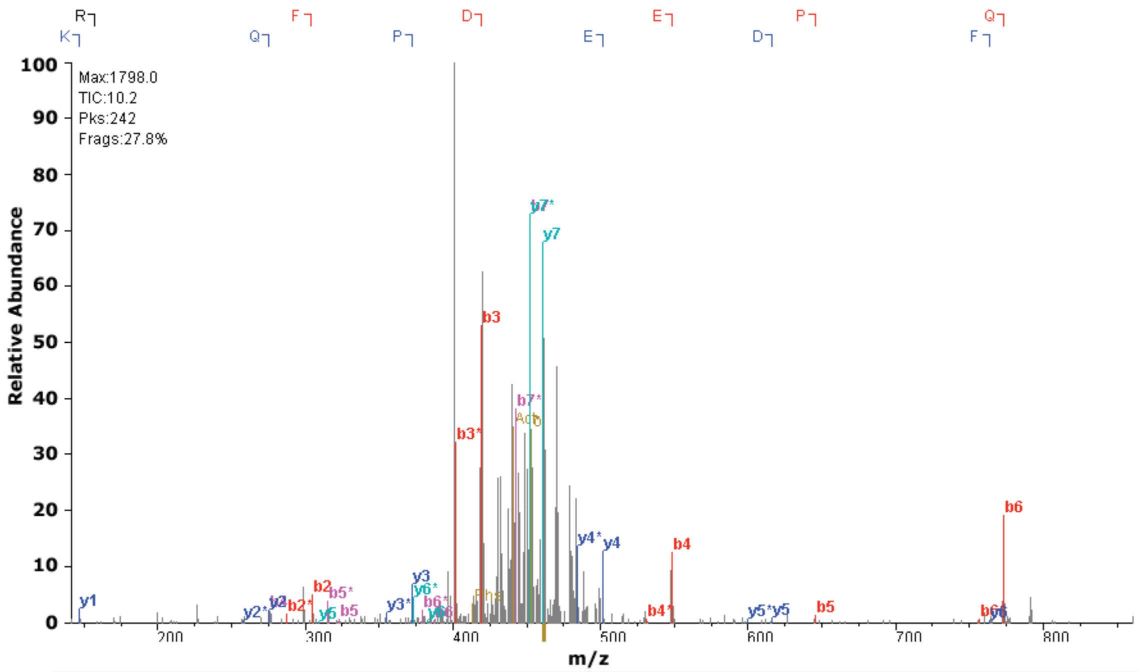

Rank/Sp	(M+H)+	XCorr	Sp	Ions	Reference	Peptide
1/231	920.01282	2.2978	3.056	10/12	AT4G27920.1	R.RFDEPQK.Y

(b)

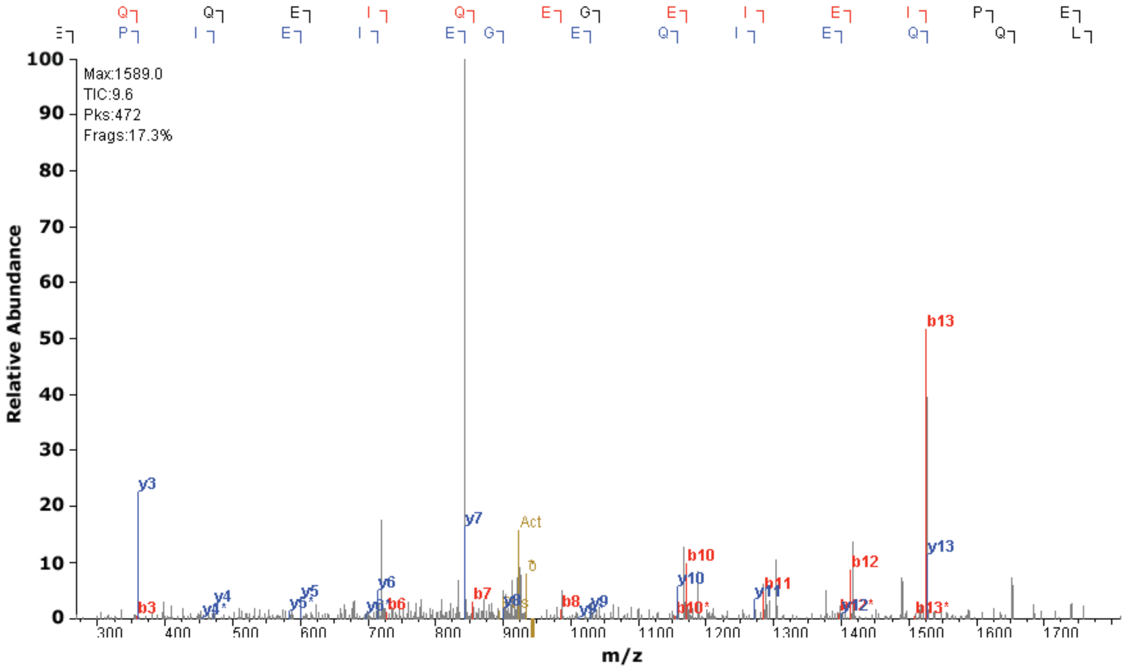

Rank/Sp	(M+H)+	XCorr	Sp	Ions	Reference	Peptide
1/1	1884.99158	2.5054	3.033	18/30	AT3G53430.1	K.DLQQEIQEGEIEIPEN.-

Figure S3 Nishimura et al.

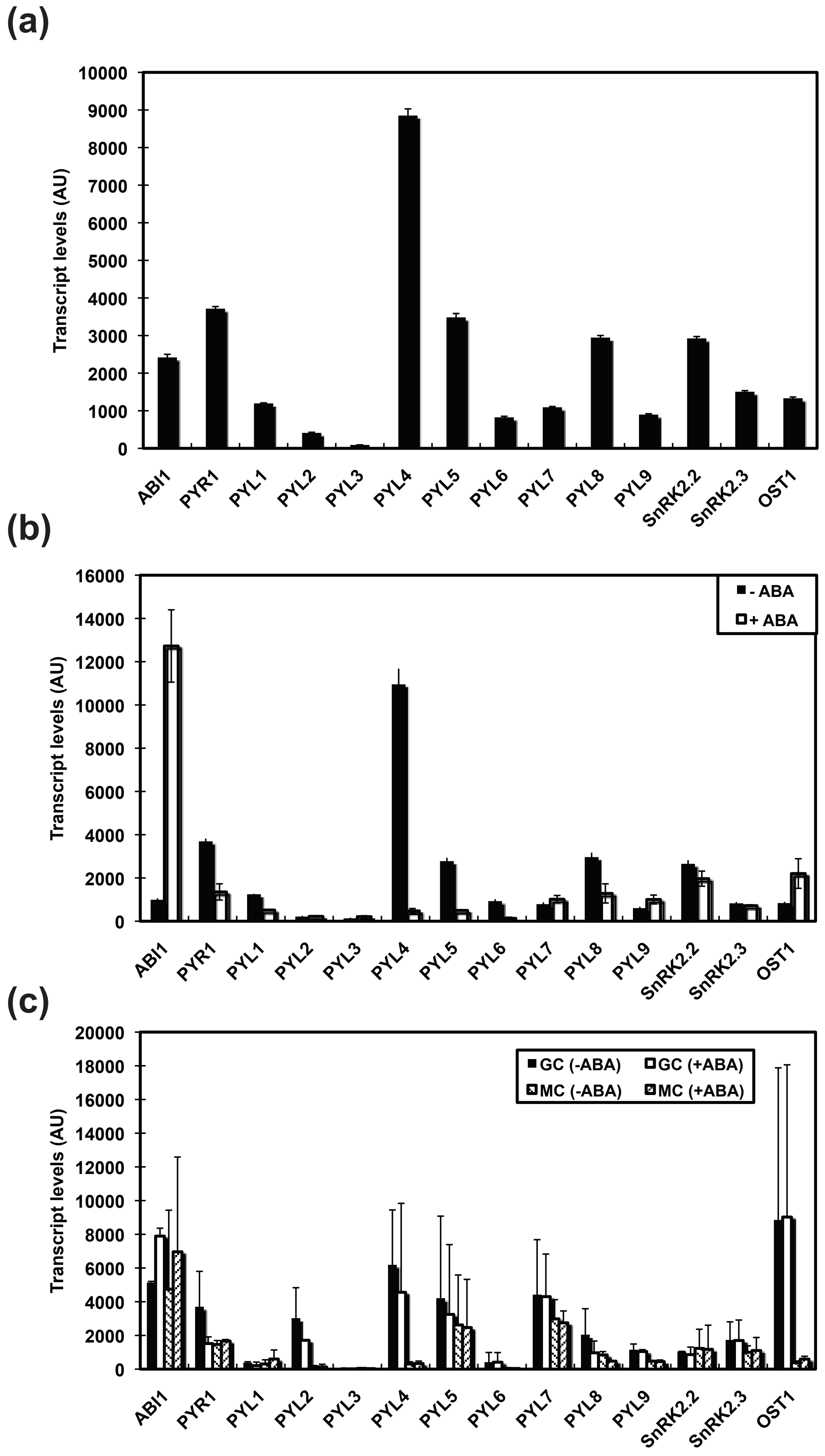

Figure S4 Nishimura et al.

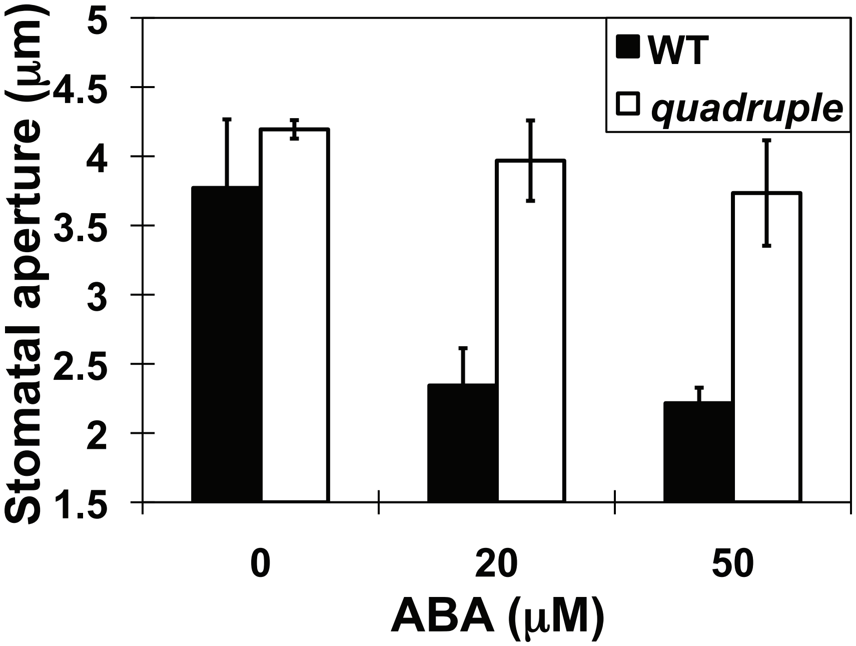

Figure S5 Nishimura et al.
