# Supplementary material for: PYR/PYL/RCAR family members are major in-vivo ABI1 protein phosphatase 2C-interacting proteins in Arabidopsis
Source: Plant J. 2009 Nov 9;61(2):290–9. doi: 10.1111/j.1365-313X.2009.04054.x (PMC2807913; doi:10.1111/j.1365-313X.2009.04054.x)
Supplement: Supplementary file 3 [file tpj0061-0290-SD5.doc]

**SI Figure legends**

**Figure S1. Identification of ABI1-interacting proteins *in Arabidopsis*.**

YFP-ABI1 and associated proteins purified from stable transgenic *Arabidopsis* plant lines expressing YFP-ABI1 or control YFP in the *abi1-3* T-DNA mutant background treated without ABA (a) and with exogenous ABA (b). Eluates from YFP control and YFP-ABI1 purifications were analyzed and visualized by silver staining. Proteins were subsequently identified by LC-MS/MS. Stars indicate predicted YFP and YFP-ABI1 bands. Arrow heads: Two of the major protein bands associated with YFP-ABI1 show molecular weights similar to SnRK2s and Bet VI protein family members.

**Figure S2. ABA-induced interaction between ABI1 and PYR1 in *Arabidopsis*.**

Total protein extracts (Input) from YFP-ABI1 and YFP plants, that were grown on MS plates for 3 weeks. Plants were incubated for 2 hours in water (-ABA samples). After pre-incubation for 2 hours in water, 3-week-old plants were treated with 100 μM ABA for 48 hours (+ABA samples). After co-immunoprecipitation using anti-GFP beads, input and immunoprecipitated samples were detected with anti-GFP and anti-PYR1 antibodies. The PYR1, ABI1-YFP, YFP and ACTIN bands are indicated by triangles. Non-specific bands are indicated by closed stars.

**Figure S3. Tandem mass spectrum of a peptides from proteins ABIP8 and ABIP11 listed in Tables S3 and S4 that showed one unique sequence to the corresponding proteins.**

The protein identifications were further supported by identified peptides that, even though their sequence are non-unique to proteins ABIP8 (a) and ABIP11 (b), non-unique peptides were not found within that of any other identified ABI1-interacting proteins that passed the DTASelect filter; thus providing further support of their link to the ABIP8 and ABIP11 proteins. Such non-unique peptide identifications (see Table S1) were fully tryptic which provides additional confidence in the correctness of the sequence match (data not shown).

**Figure S4. Transcription profiles of *ABI1*, *PYR1/PYLs* and *SnRK2s* in seedlings, guard cells and mesophyll cells.**

(a) 21 day-old seedlings, (b) 7 day-old seedlings grown on liquid medium treated with or without ABA (c) Guard cells and mesophyll cells isolated from plants treated with or without ABA. Data were obtained from (Leonhardt *et al.* 2004; Goda *et al.* 2008; Yang *et al.* 2008).

**Figure S5. ABA-induced stomatal closure is impaired in *pyr1pyl1pyl2pyl4* quadruple mutant plants.**

ABA-induced stomatal closure (20 or 50 μM [ABA]) in *pyr1pyl1pyl2pyl4* quadruple mutant and wild type abaxial leaf epidermes treated with the indicated ABA concentrations for 1 h (n=3 experiments, 30 stomata per experiment and condition; genotype and [ABA] blind experiments). Experiments in Figure S5 were performed in independent experiments from those shown in Figure 5a, confirming the strong ABA insensitivity.
