# Supplementary material for: PYR/PYL/RCAR family members are major in-vivo ABI1 protein phosphatase 2C-interacting proteins in Arabidopsis
Source: Plant J. 2009 Nov 9;61(2):290–9. doi: 10.1111/j.1365-313X.2009.04054.x (PMC2807913; doi:10.1111/j.1365-313X.2009.04054.x)
Supplement: Supplementary file 6 [file tpj0061-0290-SD6.doc]

**Table S3. The number of unique and total peptides and spectrum count of candidate ABI1-interacting proteins co-purified with ABI1 from *Arabidopsis* plants in the absence of exogenous ABA.**

|  |  |  |  | Unique peptides / Total peptides (Spectrum count) | | | | |  |
| --- | --- | --- | --- | --- | --- | --- | --- | --- | --- |
|  | Locus | AGI | Symbol | Ex1 | Ex2 | Ex3 | Ex4 | Ex5 | Average Spec count |
| ABIP1 | AT5G53160.2 | AT5G53160 | PYL8 | 14/32(1120) | 11/17(591) | 17/37(473) | 9/15(449) | 11/18(1256) | 777.8 ± 381.3 |
| ABIP2 | AT2G38310.1 | AT2G38310 | PYL4 | 21/30(385) | 8/13(145) | 18/27(161) | 6/12(46) | 10/15(99) | 167.2 ± 129.7 |
| ABIP3 | AT5G05440.1 | AT5G05440 | PYL5 | 18/21(643) | 10/12(242) | 32/35(601) | 8/9(56) | 14/14(278) | 364 ± 250.6 |
| ABIP4 | AT1G01360.1 | AT1G01360 | PYL9 | 17/20(126) | 11/11(94) | 14/17(151) | 12/15(410) | 11/13(1135) | 383.2 ± 438.6 |
| ABIP5 | AT2G40330.1 | AT2G40330 | PYL6 | 8/13(234) | 1/4(102) | 7/10(90) | 4/5(25) | 6/7(24) | 95.0 ± 85.6 |
| ABIP6 | AT4G17870.1 | AT4G17870 | PYR1 | 8/10(20) | 2/3(5) | 4/7(14) | 5/7(19) | 6/8(26) | 16.8 ± 7.9 |
| ABIP11 | AT3G53430.1 | AT3G53430 | RPL12B | 0*/4(5) | 1/2(2) | 0*/6(6) | 0*/2(2) | 0*/2(2) | 3.4 ± 2.0 |
| ABIP12 | AT5G20280.1 | AT5G20280 | ATSPS1F | 4/6(12) | 2/2(6) | 5/7(13) | 5/5(6) | 2/4(4) | 8.2 ± 4.0 |
| ABIP7 | AT4G01026.1 | AT4G01026 | PYL7 | 4/8(13) |  | 9/14(83) | 2/6(11) | 2/5(16) | 30.8 ± 34.89 |
| ABIP8 | AT4G27920.1 | AT4G27920 | PYL10 |  |  | 2/4(16) | 1/2(2) | 1/2(2) | 6.7 ± 8.1 |
| ABIP9 | AT5G46790.1 | AT5G46790 | PYL1 | 2/2(5) |  |  |  |  | 5.0 ± 0.0 |

*Genes with close homologs in the Arabidopsis genome may show no statisitcally unique peptides.

**Table S4. The number of unique and total peptides and spectrum count of candidate ABI1-interacting proteins co-purified with ABI1 from plants exposed to exogenous ABA.**

|  |  |  |  | Unique peptides / Total peptides (Spectrum count) | | | | |  |
| --- | --- | --- | --- | --- | --- | --- | --- | --- | --- |
|  | Locus | AGI | Symbol | Ex6 | Ex7 | Ex8 | Ex9 | Ex10 | Average Spec count |
| ABIP2 | AT2G38310.1 | AT2G38310 | PYL4 | 10/15(118) | 22/30(602) | 33/44(1540) | 23/29(122) | 19/21(113) | 499 ± 618.6 |
| ABIP6 | AT4G17870.1 | AT4G17870 | PYR1 | 20/23(211) | 21/26(546) | 25/29(355) | 14/15(53) | 10/10(43) | 241.6 ± 213.0 |
| ABIP1 | AT5G53160.2 | AT5G53160 | PYL8 | 9/15(103) | 15/23(689) | 15/27(368) | 11/14(85) | 6/10(23) | 253.6 ± 277.0 |
| ABIP9 | AT5G46790.1 | AT5G46790 | PYL1 | 16/17(66) | 19/22(159) | 22/24(137) | 8/8(13) | 2/2(2) | 75.4 ± 71.0 |
| ABIP4 | AT1G01360.1 | AT1G01360 | PYL9 | 3/4(30) | 15/18(274) | 19/23(173) | 9/11(29) | 6/6(10) | 103.2 ± 115.8 |
| ABIP3 | AT5G05440.1 | AT5G05440 | PYL5 | 7/7(106) | 17/17(111) | 37/40(871) | 11/11(35) | 3/3(69) | 238.4 ± 355.0 |
| ABIP7 | AT4G01026.1 | AT4G01026 | PYL7 | 3/3(3) | 1/3(6) | 10/14(120) | 17/21(76) | 8/10(25) | 46.0 ± 50.7 |
| ABIP5 | AT2G40330.1 | AT2G40330 | PYL6 | 7/7(28) | 10/12(103) | 9/14(470) | 7/8(21) | 4/4(4) | 125.2 ± 196.5 |
| ABIP13 | AT3G19390.1 | AT3G19390 | MLD14.3 | 4/5(15) | 5/5(18) | 3/3(3) | 4/4(6) | 3/3(3) | 9.0 ± 7.0 |
| ABIP14 | AT2G20580.1 | AT2G20580 | AtRPN1a | 1/2(2) | 4/6(11) | 7/9(15) | 3/3(5) | 3/3(3) | 7.2 ± 5.6 |
| ABIP15 | AT4G05420.1 | AT4G05420 | DDB1A | 0*/2(2) | 0*/6(12) | 0*/7(7) | 0*/3(4) | 0*/2(2) | 5.4 ± 4.2 |
| ABIP8 | AT4G27920.1 | AT4G27920 | PYL10 |  | 1/2(8) |  |  |  | 8.0 ± 0.0 |

*Genes with close homologs in the Arabidopsis genome may show no statisitcally unique peptides.

**Table S5. The unique sequence coverage of known ABA signaling proteins co-purified with ABI1 from *Arabidopsis*** plants without addition of exogenous ABA.

|  |  |  | Sequence coverage (%) | | | | | |
| --- | --- | --- | --- | --- | --- | --- | --- | --- |
| Symbol | Locus | AGI | Ex1 | Ex2 | Ex3 | Ex4 | Ex5 | Average |
| ABI1 | AT4G26080.1 | AT4G26080 | 84.8 | 79.8 | 78.9 | 73.1 | 75.7 | 78.5 ± 4.4 |
| SnRK2.2 | AT3G50500.1 | AT3G50500 |  |  | 12.9 |  | 4.2 | 8.6 ± 6.2 |
| SnRK2.3 | AT5G66880.1 | AT5G66880 |  |  | 1.6 | 4.1 | 7.3 | 4.3 ± 2.9 |
| RPN10 | AT4G38630.1 | AT4G38630 | 10.6 |  | 14.0 | 7.3 | 11.7 | 10.9 ± 2.8 |
| OST2 | AT2G18960.1 | AT2G18960 |  |  | 1.2 |  |  | 1.2 ± 0.0 |

**Table S6. The unique sequence coverage of known ABA signaling proteins co-purified with ABI1 from *Arabidopsis*** plants exposed to exogenous ABA.

|  |  |  | Sequence coverage (%) | | | | | |
| --- | --- | --- | --- | --- | --- | --- | --- | --- |
| Symbol | Locus | AGI | Ex1 | Ex2 | Ex3 | Ex4 | Ex5 | Average |
| ABI1 | AT4G26080.1 | AT4G26080 | 50.1 | 72.5 | 80.9 | 76.3 | 71.4 | 70.2 ± 11.9 |
| SnRK2.2 | AT3G50500.1 | AT3G50500 | 4.2 | 9.4 | 14.8 |  | 4.2 | 8.2 ± 5.1 |
| SnRK2.3 | AT5G66880.1 | AT5G66880 | 4.2 | 3.3 | 7.3 |  |  | 4.9 ± 2.1 |
| RPN10 | AT4G38630.1 | AT4G38630 | 14.5 | 11.7 | 13.7 | 7.3 |  | 11.8 ± 3.2 |
| OST2 | AT2G18960.1 | AT2G18960 |  |  | 2.8 |  |  | 2.8 ± 0.0 |

**Table S7. The unique sequence coverage of candidate ABI1-interacting proteins co-purified with ABI1 from *Arabidopsis* plants in the absence of exogenous ABA.**

|  |  |  |  | Sequence coverage (%) | | | | | |
| --- | --- | --- | --- | --- | --- | --- | --- | --- | --- |
|  | Locus | AGI | Symbol | Ex1 | Ex2 | Ex3 | Ex4 | Ex5 | Average |
| ABI1 | AT4G26080.1 | AT4G26080 | ABI1 | 84.8 | 79.8 | 78.9 | 73.1 | 75.7 | 78.5 ± 4.4 |
| ABIP1 | AT5G53160.2 | AT5G53160 | PYL8 | 36.4 | 42.9 | 40.7 | 39.2 | 41.6 | 40.2 ± 2.5 |
| ABIP2 | AT2G38310.1 | AT2G38310 | PYL4 | 70.7 | 51.3 | 43.2 | 23.8 | 48.8 | 47.6 ± 16.8 |
| ABIP3 | AT5G05440.1 | AT5G05440 | PYL5 | 66.1 | 55.2 | 67.8 | 31.3 | 57.1 | 55.5 ± 14.6 |
| ABIP4 | AT1G01360.1 | AT1G01360 | PYL9 | 60.6 | 50.3 | 39.1 | 52.1 | 51.5 | 50.7 ± 7.7 |
| ABIP5 | AT2G40330.1 | AT2G40330 | PYL6 | 42.8 | 9.0 | 37.0 | 23.9 | 30.8 | 28.7 ± 13.1 |
| ABIP6 | AT4G17870.1 | AT4G17870 | PYR1 | 34.2 | 14.6 | 24.0 | 22.0 | 39.4 | 26.8 ± 9.9 |
| ABIP11 | AT3G53430.1 | AT3G53430 | RPL12B |  | 9.2 |  |  |  | 9.2 ± 0.0 |
| ABIP12 | AT5G20280.1 | AT5G20280 | ATSPS1F | 7.2 | 4.9 | 7.3 | 5.0 | 2.1 | 5.3 ± 2.1 |
| ABIP7* | AT4G01026.1 | AT4G01026 | PYL7 | 29.7 |  | 28.7 | 10.3 | 13.7 | 20.6 ± 10.0 |
| ABIP8* | AT4G27920.1 | AT4G27920 | PYL10 |  |  | 8.7 | 3.8 | 3.8 | 5.4 ± 2.8 |
| ABIP9* | AT5G46790.1 | AT5G46790 | PYL1 | 22.6 |  |  |  |  | 22.6 ± 0.0 |

* Three additional PYR/PYL/RCAR family members identified in 4, 3 and 1 of the experiments, respectively.

Table S8. The unique sequence coverage of candidate ABI1-interacting proteins co-purified with ABI1 from plants exposed to exogenous ABA.

|  |  |  |  | Sequence coverage (%) | | | | | |
| --- | --- | --- | --- | --- | --- | --- | --- | --- | --- |
|  | Locus | AGI | Symbol | Ex6 | Ex7 | Ex8 | Ex9 | Ex10 | Average |
| ABI1 | AT4G26080.1 | AT4G26080 | ABI1 | 50.1 | 72.5 | 80.9 | 76.3 | 71.4 | 70.2 ± 11.6 |
| ABIP2 | AT2G38310.1 | AT2G38310 | PYL4 | 38.5 | 56.2 | 70.2 | 73 | 58.3 | 59.2 ± 12.2 |
| ABIP6 | AT4G17870.1 | AT4G17870 | PYR1 | 63.7 | 63.6 | 64.2 | 59.9 | 42.9 | 58.9 ± 9.1 |
| ABIP1 | AT5G53160.2 | AT5G53160 | PYL8 | 39 | 48.3 | 41.2 | 38.9 | 26.9 | 38.9 ± 7.7 |
| ABIP9 | AT5G46790.1 | AT5G46790 | PYL1 | 52.8 | 63.5 | 69.1 | 41.2 | 18.1 | 48.9 ± 20.3 |
| ABIP4 | AT1G01360.1 | AT1G01360 | PYL9 | 22.8 | 66.6 | 53.6 | 38.7 | 32.6 | 42.9 ± 17.3 |
| ABIP3 | AT5G05440.1 | AT5G05440 | PYL5 | 30.0 | 43.3 | 78.8 | 42.4 | 34.5 | 45.8 ±19.3 |
| ABIP7 | AT4G01026.1 | AT4G01026 | PYL7 | 5.2 | 52.4 | 61.4 | 42.5 | 17.5 | 35.8 ± 23.7 |
| ABIP5 | AT2G40330.1 | AT2G40330 | PYL6 | 32.1 | 37.6 | 36.6 | 28.2 | 23.3 | 31.6 ± 5.6 |
| ABIP13 | AT3G19390.1 | AT3G19390 | MLD14.3 | 12 | 21.9 | 7.3 | 13.3 | 13.3 | 13.6 ± 5.3 |
| ABIP14 | AT2G20580.1 | AT2G20580 | AtRPN1a | 1.6 | 6.9 | 7.9 | 5.6 | 5.6 | 5.5 ± 2.4 |
| ABIP8* | AT4G27920.1 | AT4G27920 | PYL10 |  | 3.8 |  |  |  | 3.8 ± 0.0 |

* PYL10 was identified in one experiment as shown.
